# *Pseudomonas aeruginosa* Takes a Multi-Target Approach to Achieve Junction Breach

**DOI:** 10.3389/fcimb.2017.00532

**Published:** 2018-01-11

**Authors:** Guillaume Golovkine, Emeline Reboud, Philippe Huber

**Affiliations:** Centre National de la Recherche Scientifique ERL5261, CEA BIG-BCI, Institut National de la Santé et de la Recherche Médicale UMR1036, Université Grenoble Alpes, Grenoble, France

**Keywords:** bacterial invasion, bacterial virulence factors, intercellular junctions, bacterial secretion systems, epithelium, endothelium

## Abstract

*Pseudomonas aeruginosa* is an opportunistic pathogen which uses a number of strategies to cross epithelial and endothelial barriers at cell–cell junctions. In this review, we describe how the coordinated actions of *P. aeruginosa*'s virulence factors trigger various molecular mechanisms to disarm the junctional gate responsible for tissue integrity.

## Introduction

Transmigration across the body's natural barriers is one of the most significant challenges for bacterial pathogens. This process is particularly challenging for opportunistic pathogens, which tend to be less armed than true pathogens. Once the bacterium has penetrated the host's external defenses it can exploit the many advantages: abundant nutrients, replicative niche and lack of bacterial competition. Although the blood is a very bactericidal environment, it can be transiently used by bacteria after entry into the body as a route to spread and thus reach the retro-endothelial compartment of many organs.

In this review, we will focus on a major opportunistic Gram-negative pathogen, *Pseudomonas aeruginosa*, responsible for nosocomial acute and chronic infections, in particular in patients with cystic fibrosis (Williams et al., 2010; Gellatly and Hancock, 2013). In chronic infections, *P. aeruginosa* colonizes the airways and forms biofilms where multiple bacteria proliferate. In acute infections, *P. aeruginosa* traverses the mucosa, provoking bacteremia and infecting distant organs.

Unless there is a breach, the skin and most multilayered mucosae (mouth, esophagus, vagina, etc.) are impenetrable to opportunistic pathogens like *P. aeruginosa*. The gut epithelium, with its extensive mucus barrier and highly competitive microbiota is also very difficult to traverse. For these reasons, bacteria mostly transmigrate at the level of the lung alveoli and the urinary tract, composed of a single layer or transitional epithelia, as well as the cornea, composed of a small number of non-keratinized cell layers (Lyczak et al., 2000; Mittal et al., 2009; Fleiszig and Evans, 2010; Williams et al., 2010). Once present in the interstitium, bacteria may reach the blood by crossing the endothelium, using specific mechanisms that will be discussed in this review.

Several mechanisms have been described by which bacterial pathogens cross the epithelium, including transcellular and paracellular (i.e., through intercellular junctions) routes and a Trojan horse strategy, where phagocytic cells are used as transporters (Doran et al., 2013). Here, we will focus on the mechanisms by which *P. aeruginosa* has been shown to traverse the epithelial and endothelial barriers.

*P. aeruginosa* is mainly an extracellular bacterium, although several groups have reported its possible internalization in non-immune cells (i.e., various epithelial cell types) *in vitro* (Fleiszig et al., 1994; Hauser et al., 1998; Kierbel et al., 2005; Zaas et al., 2009; Sana et al., 2015). However, it is not currently known whether *P. aeruginosa* is capable of traversing the epithelial layer once internalized, and no Trojan horse transmigration events have been documented so far. Several old and more recent works describe how *P. aeruginosa* alters intercellular junction components to allow its transmigration at cell–cell junctions. This body of literature suggests that the paracellular route is the main itinerary used by this bacterium to gain entry to the host (Zulianello et al., 2006; Heiniger et al., 2010; Golovkine et al., 2016a). However, before they even reach the junctions, bacteria must avoid a number of general defense mechanisms.

## The mucosae and their numerous defense mechanisms

The apical side of all mucosae are coated by a liquid layer of variable viscosity: mucus, surfactant, tears or urine. This liquid layer entraps bacteria, and flows continuously to prevent bacterial adhesion to surfaces while clearing bacteria out of the body. In the specific case of the lung, where gravity cannot assist bacterial expulsion, the muco-ciliary escalator efficiently removes bacteria from the airways. This system moves the mucus blanket in the direction of the larynx, by a mechanism promoted by the beating of cilia present on tracheal cells. In addition to the physical effects of this fluid layer surmounting epithelia, it generally contains host molecules with antimicrobial activity, such as defensins and lysozyme, as a complement to the first line of anti-bacterial defense (Williams et al., 2010; Gellatly and Hancock, 2013).

When bacteria come into contact with mucosae, a second line of defense is triggered, involving the production of pro-inflammatory cytokines and chemokines, followed by the massive recruitment of neutrophils which can internalize and eliminate bacteria thanks to phagocytic processes. LPS, flagellum, and type IV pili (pili hereafter) are efficient activators of this defense system (Williams et al., 2010).

**Figure 1:**
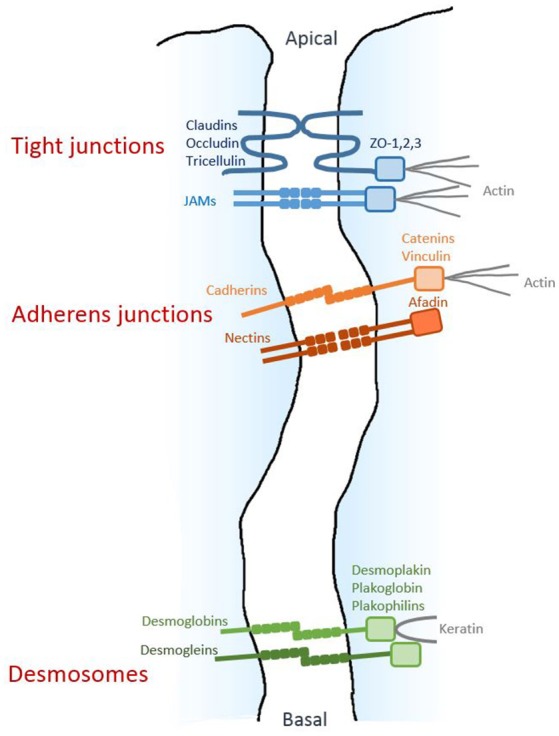
Architecture of epithelial cell-cell junctions. Diagram respresenting the three junctional structures along the junctional cleft. The homophilic adhesive proteins are shown, together with their main cytoplasmic partners.

If, despite these defensive strategies, bacteria nevertheless reach epithelial cells, two epithelial defense mechanisms further impede bacterial transmigration:

(1) The epithelial cell apical domain is refractive to toxin injection by the type III secretion system (T3SS) (Fleiszig et al., 1997; Lee et al., 1999; Kazmierczak et al., 2004), one of *P. aeruginosa's* most potent virulence factors (see below).

(2) The integrity of the epithelial barrier is ensured by several junctional structures located in the intercellular space (Farquhar and Palade, 1963; Cereijido et al., 2004); these structures are described below (Figure 1).

Tight junctions (TJs), located near the apical side of the epithelium, are composed of a branching network of close contacts between neighboring cells, forming a continuous junction (“zonula”) at the cellular periphery and establishing a “gate” between the apical and basal compartments. The transmembrane proteins mediating homophilic adhesion at TJ are members of the claudin family, as well as occludin, tricellulin and the junctional adhesion molecule (JAM) proteins. Their intracellular domains interact with ZO-1, -2 and -3 proteins, which constitute molecular platforms for various cytosolic components, including actin filaments.

The adherens junctions (AJs), located more basally, also form a zonula mainly composed of E-cadherin transmembrane proteins clustering in *cis* and promoting homophilic adhesion in *trans* with E-cadherin molecules on adjacent cells. Inside the cell, these junctions are linked to the actin cytoskeleton by catenins and vinculin. The adhesive activity of the AJ complex is fine-tuned by tyrosine- and serine/threonine-kinases, as well as Rac and Rap1 GTPases (Boettner and Van Aelst, 2009; Serrels et al., 2011; McCole, 2013; Ratheesh et al., 2013). Nectin is another transmembrane protein with homophilic adhesive activity; it is located in AJs and is linked to actin by afadin. Importantly, the presence of AJs is required for the formation and maintenance of the other junctional structures.

Finally, closer to the basal side of the epithelium, the desmosomes form spots (“macula”) along the cell's circumference. These macula are composed of adhesive proteins desmoglein and desmoglobin, which are linked to keratin filaments by desmoplakin, plakoglobin, and plakophilins.

While both TJs and AJs are targets of *P. aeruginosa*'s virulence factors (see below), so far, no toxic action of *P. aeruginosa* on desmosomes has been reported.

All of these multi-bolt junctional structures work together to provide strong interactions between epithelial cells and strictly control paracellular permeability to molecules and cells.

In normal settings, epithelia are thus protected from bacterial infiltration by an array of mechanisms. However, after certain physical, chemical or biological insults, the priming effects of which have yet to be completely characterized *in vivo*, mucosae can become permissive to bacteria.

## Initial stages of *P. aeruginosa*-mucosa interaction

Before penetration, bacteria must first reach a site compatible with transmigration. A polar flagellum is present in most *P. aeruginosa* strains. This structure allows the bacteria to swim in liquids and rapidly scan cellular surfaces for transmigration opportunities (Luzar et al., 1985). In this context, *P. aeruginosa* investigates the cell surface by employing several types of swimming trajectories (Golovkine et al., 2016b). Recent findings indicate that an active flagellum is indeed required for efficient infection *in vivo* (Turner et al., 2014).

Another type of motion is provided by pili. Pili are thin filaments which extend and retract through filament assembly and disassembly in bacterium's inner membrane (Leighton et al., 2015). The tip of the pilus displays adhesive properties allowing bacteria to interact with abiotic and cellular surfaces. By retracting attached pili and detaching others, *P. aeruginosa* can engage in a type of surface motility by twitching movement.

Both swimming and twitching motions allow bacteria to investigate the mucosa and to resist the host's flushing systems.

Bacteria bound to the epithelial surface can also remain immobile and form aggregates of tens to hundreds of individuals embedded in a biofilm-like matrix (Lepanto et al., 2011). This bacterial aggregate can locally alter the apical surface of the host cell, transforming it into an area with basolateral-domain composition (Kierbel et al., 2007). The transformed area can then recruit AJ proteins, like E-cadherin/β-catenin and afadin. A similar process has been reported for *Neisseria meningitidis* at the apical surface of the blood-brain barrier, which eventually leads to junction attrition (Coureuil et al., 2009). However, for *P. aeruginosa*, this process does not disrupt intercellular junctions (Kierbel et al., 2007; Tran et al., 2014).

**Figure 2:**
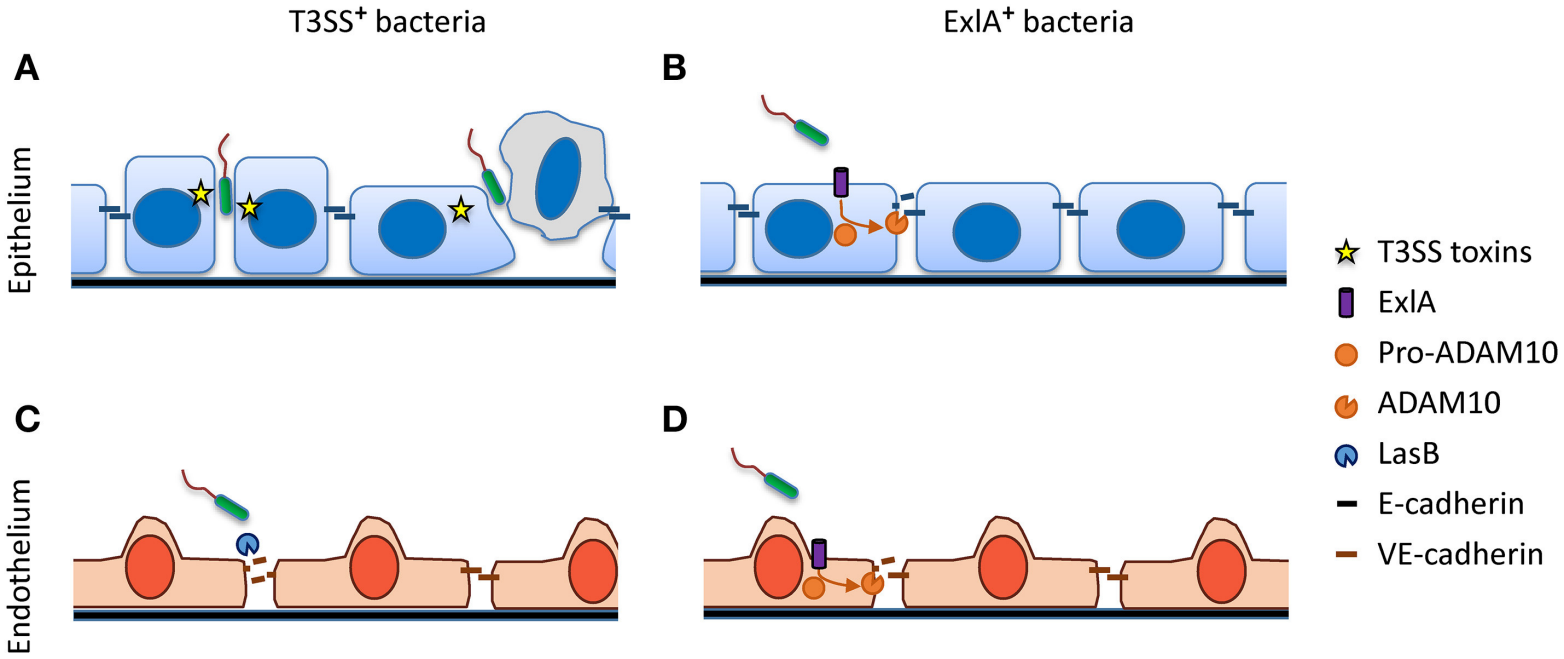
Disruption of adherens junctions by *P. aeruginosa*. **(A)** T3SS+ bacteria exploit transient junctional weakenings, occurring during cell division or senecent cell exclusion, to penetrate the epithelial junction cleft. **(B)** Pore formation induced by ExlA+ bacteria activates the metalloprotease ADAM10, which in turn cleaves E-cadherin. **(C)** Most T3SS+ bacteria secrete the protease LasB, which cleaves endothelial VE-cadherin. **(D)** Similar to epithelial cells, ExlA induces VE-cadherin cleavage via ADAM10 activation in endothelial cells.

As mentioned above, studies with *in vitro* and *ex vivo* epithelial systems converge toward a mechanism in which bacterial transmigration occurs at cell–cell junctions. However, not all junctions are permissive to bacterial transmigration; transmigration therefore remains a rare event in reconstituted polarized epithelia, and probably *in vivo* as well. Our group recently showed that some *P. aeruginosa* strains take advantage of naturally occurring mini-breaches in epithelial cell–cell junctions, either during cell division or when senescent cells are expelled from the epithelial layer (Figure 2A) (Golovkine et al., 2016a). In both situations, a transient loss of E-cadherin at cell–cell junctions allows infiltration of one bacterium, followed by a cohort of other bacteria attracted by chemotaxis to the same entry-point. Therefore, tissue repair mechanisms may increase the capacity of *P. aeruginosa* to cross the epithelial layer.

## Bacterial virulence factors involved in junction disruption

*P. aeruginosa* synthesizes a number of regulated virulence factors which work synergistically in the context of infection. In this review, we focus on factors that have been demonstrated to play a pivotal role in bacterial transmigration across epithelial and endothelial barriers. Their various actions on junctions are summarized in Table 1. Further information about *P. aeruginosa* virulence factors can be found in several excellent reviews (Hauser, 2009; Bleves et al., 2010; Sawa, 2014).

**Table 1:** *P. aeruginosa* virulence factors altering intercellular junctions.

	**Virulence factors**	**Effects**	**References**
General effects on transmigration	T2SS: LasB	Increased MP and JD	Azghani, 1996; Azghani et al., 2000; Nomura et al., 2014
	QS: 3O-C12-HSL	Increased MP and JD	Vikström et al., 2009; Halldorsson et al., 2010; Schwarzer et al., 2014
	T3SS: ExoS	Increased MP, cell rounding ExoS action confirmed *in vivo* in lung infection	Pederson et al., 1999; Fraylick et al., 2001; Soong et al., 2008; Ganter et al., 2009; Huber et al., 2014 Rangel et al., 2015
	T3SS: ExoT	Cell rounding	Garrity-Ryan et al., 2000; Kazmierczak and Engel, 2002; Ganter et al., 2009; Huber et al., 2014
	Type 4 pili	Required for T3SS-dependent toxicity and bacterial transmigration	Heiniger et al., 2010; Hayashi et al., 2015; Golovkine et al., 2016a
	Flagellum	Required for bacterial transmigration	Golovkine et al., 2016a
	TPS: ExlA	Increased MP and JD	Elsen et al., 2014; Bouillot et al., 2017; Reboud et al., 2017
	T2SS: ToxA	Increased MP and JD	Azghani, 1996
	Rhamnolipids	Increased MP and JD Induction of paracellular transmigration	Graham et al., 1993; Zulianello et al., 2006; Halldorsson et al., 2010; Wallace et al., 2014
Effect on the actin cytoskeleton	T3SS: ExoS, ExoT	Actin filament depolymerization via inactivation of Rho GTPases	Garrity-Ryan et al., 2000; Kazmierczak and Engel, 2002; Soong et al., 2008; Huber et al., 2014
Effects on tight junctions	T2SS: LasB	Decreased cellular levels of occludin, claudin-1 and 4, tricellulin	Beaufort et al., 2013; Nomura et al., 2014
		Unaltered expression of claudin-5 and -7, ZO-1 and -2 in nasal epithelial cells	Nomura et al., 2014
		Decreased ZO-1 and−2 levels in MDCK	Azghani, 1996
	QS: 3O-C12-HSL	Decreased levels and Tyr-hyperphosphorylation of ZO-1, ZO-3, and JAM-A Dephosphorylation of occludin Decreased interaction between junction components	Vikström et al., 2009, 2010
	T3SS: ExoS	ZO-1 and occludin localization altered by ExoS	Soong et al., 2008
Effects on adherens junctions	T2SS: LasB	VE-cadherin cleavage	Beaufort et al., 2013; Golovkine et al., 2014
		E-cadherin and ß-catenin levels unaltered	Azghani et al., 1993; Golovkine et al., 2014; Nomura et al., 2014
	QS: 3O-C12-HSL	Decreased E-cadherin and ß-catenin expression Tyr hyperphosphorylation of both proteins Ser/Thr-hyperphosphorylation of E-cadherin Ser/Thr dephosphorylation of ß-catenin	Vikström et al., 2009
	T3SS: ExoS, ExoT	Adherens junction disorganization due to Rac inactivation	Huber et al., 2014
	TPS: ExlA	E- and VE-cadherin degradation	Reboud et al., 2017
	LecB lectin	ß-catenin degradation	Cott et al., 2016

*3O-C12-HSL, N-(3-oxododecanoyl)-L-homoserine lactone; JD, junction disruption; MP, monolayer permeability; QS, quorum sensing; T2SS, type II secretion system; T3SS, type III secretion system; TPS, two-partner secretion*.

### LasB protease

LasB is a potent protease with broad substrate specificity. It is secreted into the extracellular medium by most *P. aeruginosa* strains through their T2SS (Bleves et al., 2010). LasB increases epithelial monolayer permeability and induces junction disruption by downregulating the expression of several TJ components: occludin, claudin-1 and−4, and tricellulin (Azghani et al., 1993, 2000; Beaufort et al., 2013; Nomura et al., 2014), without affecting E-cadherin in AJ (Azghani et al., 1993; Golovkine et al., 2014; Nomura et al., 2014). In response to LasB, decreased levels of ZO-1 and−2 have also been noted in the MDCK epithelial cell line, whereas no difference in their expression level was detected in nasal epithelial cells grown *ex vivo* (Azghani, 1996; Nomura et al., 2014). Several signaling proteins are involved in the transient downregulation of TJ protein expression, including protein kinase C, the MAP kinases (ERK, P38 and JNK), phosphatidylinositol 3-kinase, the cyclo-oxygenase-1 and -2 and the protease activated receptor-2 (Clark et al., 2011; Nomura et al., 2014). However, how these signaling proteins are altered by LasB is essentially unknown and their interplay with the TJ components remain elusive.

### Quorum sensing

The expression of LasB and several other bacterial proteins is controlled by quorum sensing (QS), a system of bacterial communication based on cell density (Schuster et al., 2003). One of the QS signaling molecules produced by bacteria, N-(3-oxododecanoyl)-L-homoserine lactone (3O-C12-HSL), can act directly on host TJs. Exposure to 3O-C12-HSL induces a decrease in the cellular contents and tyrosine-hyperphosphorylation of ZO-1, ZO-3 and JAM-A, and simultaneously triggers occludin dephosphorylation (Vikström et al., 2009, 2010). These modifications lead to a loss of interaction between TJ components, resulting in the transient loss of TJ sealing capacity (Vikström et al., 2009; Halldorsson et al., 2010; Schwarzer et al., 2014). Incubation of epithelial cells with 3O-C12-HSL also decreases cellular levels of E-cadherin/β-catenin and modifies their phosphorylation status, diminishing homophilic binding (Vikström et al., 2009).

### The type III secretion system

As mentioned above, *P. aeruginosa*'s T3SS delivers exotoxins to the cytoplasm of host cells (Deng and Barbieri, 2008; Hauser, 2009). Four toxins have been identified: ExoU, ExoS, ExoT, and ExoY. In general, the different genes coding for exotoxins are not all present in a single bacterial clone; in particular, *exoU* and *exoS* are mutually exclusive. ExoU is a phospholipase inducing plasma membrane disruption and necrotic cell death. Therefore, it promotes bacterial transmigration by killing epithelial cells. ExoY displays nucleotidyl cyclase activity resulting in microtubule and actin fiber alterations, but its toxic activity remains elusive. ExoS and T, present in most hospital strains, have been the subjects of more extensive study. These two toxins display GTPase-activating protein activity, targeting Ras-, Rho- and Rab-family GTPases through their ADP-ribosyl-transferase activity (ExoS) or the focal contact proteins Crk1-2 (ExoT). In particular, inactivation of Rho GTPases (i.e., Rho, Rac and cdc42) by ExoS or ExoT induces the dephosphorylation of Lim kinase and subsequently of cofilin (Huber et al., 2014). Cofilin is an actin depolymerizing enzyme which is active in its unphosphorylated state. Hence, ExoS and ExoT, via inactivation of Rho GTPases, cause actin cytoskeleton depolymerization in all intracellular locations, depriving AJs and TJs of an intracellular component essential for their full adhesive capacity. Rho and Rac are also required at AJs to promote protein complex association and strong cell–cell interaction (Yamada and Nelson, 2007). The inhibition of Rho and Rac by ExoS/ExoT may thus directly affect the organization of the AJ complex. Interestingly, the pharmacological activation of another GTPase, Rap1, which also drives AJ complex organization, was shown to counteract the effect of ExoS/ExoT on junction disruption and to reduce exotoxin-induced cell rounding (Bouillot et al., 2015). In addition, ExoS has been shown to use unexplored mechanisms to displace ZO-1 and occludin from the TJ (Soong et al., 2008).

### Type IV pili

Toxin injection by the T3SS requires the presence of pili. Tension in the pilus induced by surface attachment triggers the synthesis of various virulence factors, including the T3SS (Persat et al., 2015; Inclan et al., 2016). Pili are also essential for the close bacterium-host cell interaction required for T3SS toxin injection (Heiniger et al., 2010; Hayashi et al., 2015). Pili thus have a major impact on bacterial transmigration (Heiniger et al., 2010; Hayashi et al., 2015; Golovkine et al., 2016a) in addition to their motility-inducing capacity, and probably each of their functions are essential to this process.

Together, the T3SS, the pili and the flagellum are all required for transmigration across epithelial cell junctions (Heiniger et al., 2010; Hayashi et al., 2015; Golovkine et al., 2016a). However, the T3SS effectors are only injected into host cells through their baso-lateral membrane domain, i.e., when bacteria penetrate the cell layer or when they are in the sub-epithelial compartment (Fleiszig et al., 1997; Lee et al., 1999; Kazmierczak et al., 2004). As a consequence, the T3SS is probably not the primary virulence factor required for bacterial transmigration across mucosae. It is nevertheless actively involved in acute infections, as elegantly shown in infected mouse lungs (Rangel et al., 2015).

### ExlA

Recently, a pore-forming toxin named Exolysin, or ExlA, was identified in a subset of *P. aeruginosa* outlier strains lacking T3SS (Elsen et al., 2014; Huber et al., 2016; Reboud et al., 2016). ExlA is a cytolysin, secreted by a two-partner secretion (TPS) system, that forms a 1.6-nm diameter pore in the host plasma membrane (Basso et al., 2017). ExlA induces E-cadherin cleavage by a multistep, subtly controlled mechanism (Figure 2B) (Reboud et al., 2017). The cadherins are natural substrates of the transmembrane metalloprotease ADAM10 (Pruessmeyer and Ludwig, 2009). Its precursor form, pro-ADAM10, is maintained in an inactive state through interaction with calmodulin, a cytosolic protein which has a high affinity for Ca^2+^. ExlA pore formation triggers a massive Ca^2+^ influx into the cytosol, which results in the dissociation of calmodulin and pro-ADAM10. ADAM10 can then be activated through cleavage by furin. In turn, E-cadherin is rapidly cleaved by mature ADAM10, provoking junction disruption (Reboud et al., 2017). Thus, similar to α-hemolysin (Hla), a pore-forming toxin from *Staphylococcus aureus* (Inoshima et al., 2011), ExlA can subvert a highly regulated host mechanism involved in cadherin shedding to efficiently disrupt cell–cell junctions.

### Other factors

The lectin LecB, located at the surface of *P. aeruginosa*, also targets epithelial AJs, inducing β-catenin degradation by unknown mechanisms (Cott et al., 2016). The exotoxin A (ToxA or ETA), secreted by the T2SS, inhibits host protein synthesis by ADP-ribosylating elongation factor-2 (Bleves et al., 2010). Exposure to ToxA results in increased monolayer permeability and junction disruption, possibly due to interruption of protein translation (Azghani, 1996). Finally, the rhamnolipids produced by *P. aeruginosa* also appear to be important in the transmigration process, but their mechanisms of action remain to be elucidated (Graham et al., 1993; Zulianello et al., 2006; Halldorsson et al., 2010; Wallace et al., 2014).

From this overview, it emerges clearly that *P. aeruginosa* has developed multiple, highly-targeted virulence strategies affecting the TJs and the AJs to achieve transmigration.

## The specific case of the vascular endothelium

The endothelium is the innermost tunica of all vessel types. It is composed of a single thin layer of endothelial cells with cobblestone morphology, and it is endowed with the barrier properties of the vascular system. *P. aeruginosa* transmigration across the endothelium has been investigated in a small number of studies (Ganter et al., 2009; Vasil et al., 2009; Elsen et al., 2014; Golovkine et al., 2014; Huber et al., 2014). Like for epithelial cells, the apical domain of endothelial cells is refractory to injection with the T3SS, whereas their baso-lateral domain is permissive. The intercellular junctions are organized similarly to epithelial junctions, except that no desmosome is present and AJs and TJs are intermingled (Wallez and Huber, 2008). AJs contain an endothelial-specific cadherin, VE-cadherin, and TJs exhibit a different group of claudins. In contrast to E-cadherin, VE-cadherin is sensitive to LasB protease, which cleaves the protein within its extracellular part to release the adhesive domain (Figure 2C) (Beaufort et al., 2013; Golovkine et al., 2014). Thus, LasB is probably *P. aeruginosa*'s master virulence factor, orchestrating its transmigration across endothelia. Once bacteria are present in the intercellular space or underneath the cell, the T3SS and its effectors can effectively induce cellular retraction by dismantling the actin cytoskeleton, further disrupting the endothelial barrier. Like in epithelial cells, VE-cadherin is rapidly cleaved by ExlA-secreting bacteria through a mechanism involving ADAM10 (Figure 2D) (Reboud et al., 2017).

## Breaching the basement membrane

The basement membrane is a layer of extracellular matrix synthesized by epithelial and endothelial cells. This membrane, composed of laminins and type IV collagen, is in direct contact with the basal side of these cells. Both LasB and AprA, another protease secreted by *P. aeruginosa*, can degrade laminins (Heck et al., 1986a), whereas only LasB efficiently cleaves type IV collagen (Heck et al., 1986b; Bejarano et al., 1989). *P. aeruginosa* is thus equipped to readily digest and cross this layer. Other matrix proteins found in connective tissues, including type III collagen, fibronectin, vitronectin and elastin can also be cleaved by *P. aeruginosa* proteases (Heck et al., 1986b; Beaufort et al., 2011; Reboud et al., 2016) to allow bacterial progression in the mesenchymal compartment.

## Conclusion and future prospects

A number of molecular mechanisms have been reported to explain how *P. aeruginosa* transmigrates across tissue barriers, and a multistep process can be proposed involving the cooperation of several virulence factors at different stages during bacterial penetration. However, these mechanisms were determined in cellular models of infection, and very few *in vivo* confirmations are available, except for the presence of host protein degradation products and the injection of exotoxins into cells within host tissues. *In vivo* bacterial transmigration may be influenced by tissue architecture, fluid flow and the immune system. The effects of these factors cannot be fully or easily modeled through reductionist approaches. Intravital microscopy can now be used in lungs, and this approach will be instrumental in helping us to understand how acute infection proceeds in this organ (Masedunskas et al., 2012). In particular, it should provide a better appreciation of the priming steps leading to barrier permissivity. Furthermore, the possibility to observe bacteria by intravascular microscopy (Broadley et al., 2016) should provide information on the fate and extravasation of *P. aeruginosa* in bacteremia.

## Author contributions

All authors contributed to the writing of various parts of the review. PH: made Table 1 and Figure 1; GG: made Figure 2; All authors reviewed the manuscript, the table and the figures.

### Conflict of interest statement

The authors declare that the research was conducted in the absence of any commercial or financial relationships that could be construed as a potential conflict of interest.

## Abbreviations

3O-C12-HSL: N-(3-oxododecanoyl)-L-homoserine lactone
AJ: adherens junction
QS: quorum sensing
T2SS: Type II secretion system
T3SS: Type III secretion system
TJ: tight junction.
