# Corrigendum: *Pseudomonas aeruginosa* Takes a Multi-Target Approach to Achieve Junction Breach

**DOI:** 10.3389/fcimb.2018.00052

**Published:** 2018-03-09

**Authors:** Guillaume Golovkine, Emeline Reboud, Philippe Huber

**Affiliations:** Centre National de la Recherche Scientifique ERL5261, CEA BIG-BCI, Institut National de la Santé et de la Recherche Médicale UMR1036, Université Grenoble Alpes, Grenoble, France

**Keywords:** bacterial invasion, bacterial virulence factors, intercellular junctions, bacterial secretion systems, epithelium, endothelium

In the original article, part of Figure 1 was reproduced from a previous publication without copyright agreement. The modified version of Figure 1, excluding the transmission electronic micrograph, appears below together with a new figure legend, and reference Rhodin (1974) has been removed. The authors apologize for this illegitimate inclusion. This modification does not change the scientific conclusions of the review in any way.

**Figure 1:**
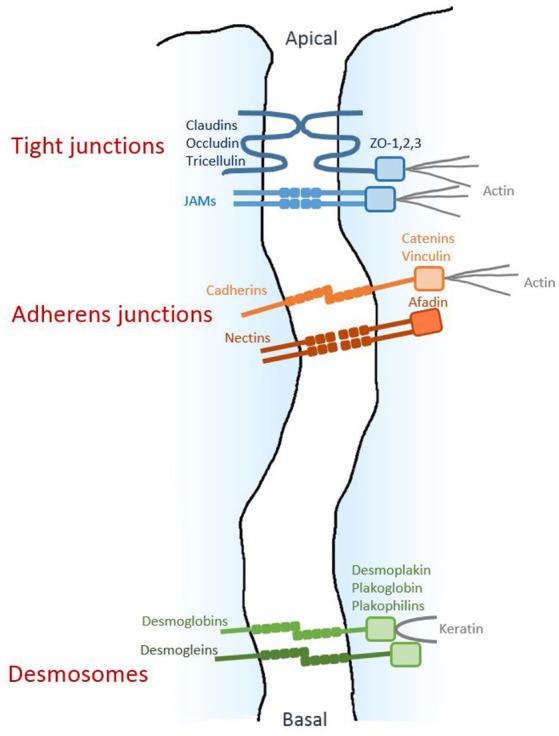
Architecture of epithelial cell-cell junctions. Diagram respresenting the three junctional structures along the junctional cleft. The homophilic adhesive proteins are shown, together with their main cytoplasmic partners.

The original article has been updated.

## Conflict of interest statement

The authors declare that the research was conducted in the absence of any commercial or financial relationships that could be construed as a potential conflict of interest.
